# IMRT and brachytherapy comparison in gynaecological cancer treatment: thinking over dosimetry and radiobiology

**DOI:** 10.3332/ecancer.2019.993

**Published:** 2019-12-17

**Authors:** Valentina Pinzi, Valeria Landoni, Federica Cattani, Roberta Lazzari, Barbara Alicja Jereczek-Fossa, Roberto Orecchia

**Affiliations:** 1 Department of Neurosurgery, Radiotherapy Unit, Fondazione IRCCS Istituto Neurologico Carlo Besta, 20133 Milan, Italy; 2 Laboratory of Medical Physics and Expert System, IRCCS Istituto Nazionale Tumori Regina Elena, 00128 Rome, Italy; 3 Unit of Medical Physics, European Institute of Oncology IRCCS (IEO), 20141 Milan, Italy; 4 Department of Radiation Oncology of IEO, European Institute of Oncology IRCCS, 20141 Milan, Italy; 5 Department of Oncology and Hemato-Oncology of University of Milan, 20122 Milan, Italy; 6 Scientific Directory of IEO, European Institute of Oncology IRCCS, 20141 Milan, Italy

**Keywords:** cervical cancer, endometrial cancer, brachytherapy, IMRT, radiobiology, dosimetry

## Abstract

**Background:**

The role of radiotherapy and brachytherapy in the management of locally advanced cervical and endometrial cancer is well established. However, in some cases, intracavitary brachytherapy (ICBRT) is not recommended or cannot be carried out. We aimed to investigate whether external-beam irradiation delivered by means of intensity-modulated radiation therapy (IMRT) might replace ICBRT in gynaecological cancer when the standard ICBRT boost delivering cannot be administered for technical or clinical reasons.

**Materials and methods:**

Fifteen already delivered treatments for gynaecological cancer patients were analysed. The treatments were performed through 3-dimensional conformal radiotherapy (3D-CRT) to the whole-pelvis up to the dose of 45–50.4 Gy followed by a boost dose administered with ICBRT in high-dose-rate or pulsed-dose-rate modality. For each patient, IMRT plans were elaborated to mimic the ICBRT. We analysed the ICBRT boost versus IMRT boost in terms of dosimetric and radiobiological aspects.

**Results:**

Mean conformity index value calculated on boost volume was 0.73 for ICBRT and 0.97 for IMRT. Mean conformation number was 0.24 for ICBRT boost and 0.78 for IMRT boost. Mean normal tissue complication probability (NTCP) values for 3D-CRT plus ICBRT and for IMRT (pelvis plus boost) were, respectively, 28% and 5% for rectum; 1.5% and 0.1% for urinary bladder and 8.9% and 6.1% for bowel.

**Conclusions:**

Our findings suggest that IMRT may represent a viable alternative in delivering the boost in patients diagnosed with gynaecological cancer not amenable to ICBRT.

## Introduction

As stated by the International Federation of Gynecology and Obstetrics (FIGO) [1], the role of radiotherapy and brachytherapy in the management of locally advanced cervical and endometrial cancer is well established. According to the clinical and radiological stage, radiation therapy either with or without concurrent chemotherapy followed by brachytherapy [2] is used as exclusive or postoperative treatment. Irradiation is usually delivered by whole-pelvic external beam radiotherapy (EBRT) ± lomboaortic tract, followed by intracavitary brachytherapy (ICBRT). Selected limited cases can be treated with exclusive pulsed dose rate (PDR) ICBRT. While locally advanced cervical cancer is treated with exclusive EBRT plus ICBRT and chemotherapy, endometrial cancer is generally treated with a surgical approach and the indication to radiotherapy depends on the stage and other histological findings. In some early stages, ICBRT can be the only treatment, while in higher stages, EBRT can be associated. Vaginal vault recurrence in endometrial cancer can be treated with ICBRT alone. In some cases, ICBRT is not recommended or cannot be carried out not only for technical limitations (i.e. difficulty in cannulating the cervix) but also for the rapid fall-off of the dose in challenging scenarios, like bulky disease, irregular geometry of tumour or medical and logistic reasons [3, 4]. Some authors have challenged the use of ICBRT by using EBRT [4–8]. Intensity-modulated radiation therapy (IMRT) can be an alternative approach to deliver a high dose to irregular and concave target volumes while reducing the volume of normal tissues irradiated. Moreover, some studies have already shown that IMRT can substitute ICBRT for boost dose delivery [6–10]. Likewise, the use of IMRT for whole-pelvis irradiation is now widely used and a decreased toxicity to the bowel and other critical structures has been reported with this technique [11–16]. Indeed, IMRT allows performing dose escalation with comparable side effects due to the possibility of a better sparing of organs at risk (OARs) [14, 15, 17].

The aim of this study is to investigate the use of IMRT for the boost treatment as an alternative to ICBRT in terms of dosimetric and radiobiological parameters for gynaecological cancers. We also compared the 3D-CRT plus ICBRT to IMRT pelvis plus boost plans.

## Patients and methods

### Patients and treatment characteristics

The patient population consists of 15 selected patients with histological proven cervical or endometrial cancer, with clinical or pathological stage IB2 bulky–IVA (FIGO stage) [1], treated at the Division of Radiotherapy of the European Institute of Oncology, Milan, Italy, before February 2006. The characteristics of patients are summarised in Table 1.

Clinical and dosimetric data of the patients were retrospectively reviewed.

### Treatment planning procedures

All patients were treated with adjuvant or exclusive whole-pelvis 3D-CRT followed by ICBRT boost.

All patients underwent computed tomography (CT)-based planning with custom immobilisation in the supine position. Clinical target volume (CTV) consisted of tumour volume, also named as gross tumour volume (GTV) plus a margin of 5–7 mm and regional lymph nodes for exclusive treatment, or tumour bed for postoperative treatment. The tumour bed consisted of cervix, uterus, parametria and upper part of the vagina in cervical cancer, vaginal vault in endometrial cancer. The planning target volume (PTV) for the pelvis (PTV_pelvis_) was obtained adding a 7–10 mm margin to the CTV [9, 11, 12, 18]. Normal tissues included the rectum wall, urinary bladder wall, bowel and femoral heads.

### Current study

All procedures were in accordance with the ethical standards and with the Helsinki Declaration of 1975.

For the purpose of this study, two treatment plans were prepared for each patient: (a) 3D-CRT plus ICBRT treatment and (b) IMRT (pelvis plus boost) treatment.

(a) 3D-CRT plus ICBRT treatment

The radiation course was administered in two steps: a whole-pelvis 3D-CRT with a four-fields-box technique followed by ICBRT boost. 3D-CRT plans were generated using a commercial treatment planning system (TPS) (Eclipse v.8.6 Varian). The 3D-CRT consisted of four-fields-box plans obtained using 18 MV photons. Anterior, posterior, right lateral and left lateral directions were used.

For the ICBRT boost, patients were equally divided in to three groups, as follows: the first group (I) consisted of five patients who underwent postoperative pelvic 3D-CRT and high-dose-rate (HDR) ICBRT boost (Micro-Selectron HDR; Nucletron Int. B.V., Veenendaal, The Netherlands) using an Ir-192 source Fletcher-Suit-Delclos applicator with intravaginal ovoids. The second group (II) consisted of five patients who underwent postoperative pelvic 3D-CRT and PDR ICBRT boost (Micro-Selectron PDR; Nucletron Int. B.V., Veenendaal, The Netherlands) with Fletcher-Suit-Delclos applicator. The third group (III) consisted of five patients who underwent radical pelvic 3D-CRT and PDR ICBRT boost with complete Fletcher-Suit-Delclos applicator with an intrauterine probe. The irradiation boost schedules were as follows: 15 Gy in three fractions for groups I and II, and 15 or 30 Gy continuously with PDR approach for group III. The dose for each impulse was 0.5 Gy for each PDR treatment.

The ICBRT boost was delivered 2 or 3 weeks after the end of the 3D-CRT. For ICBRT planning, a bladder Foley catheter and a radio-opaque rectal probe were used, and a standard Fletcher-Suit-Delclos intracavitary applicator was inserted. A post-implant contrast medium CT-scan was performed. The boost volumes for ICBRT (BOOST_ICBRT_) consisted of the vaginal vault and/or cervix and parametria plus a 5-mm margin. Rectum wall, urinary bladder wall, bowel and femoral heads were delineated. All volumes were contoured by the same radiation oncologist. An A CT-based 3D dose planning system was used for ICBRT planning (PLATO, Nucletron Int. B.V. Veenendaal, The Netherlands).

(b) IMRT treatment

For the purpose of this study, an IMRT treatment has been planned for each patient.

Each plan was originally optimised and calculated by using an Eclipse (Varian Inc.) TPS. Treatments were simulated with 15-MV linear accelerator equipped with a Millennium multileaf collimator (120 leaves) by a sliding window technique. The dose resolution grid used to calculate the dose distribution was set at 0.5 × 0.5 × 0.5 cm^3^.

The plans included pelvic IMRT followed by sequential IMRT boost. Prescription doses were the same as 3D-CRT for the pelvis, while boost doses were equivalent to the ICBRT doses in terms of biologically effective dose (BED) (*α*/*β* = 10 Gy). We contoured on the same CT images already used for the 3D-CRT treatment. The boost volumes consisted of the vaginal vault and/or cervix and parametria plus a 7–10 mm margin for IMRT (BOOST_IMRT_); the added margin was larger than the one applied for ICBRT to take into account the set-up errors and organs motion [19, 20].

In Table 2, the OARs and target volumes for the two treatment techniques are reported.

### Treatment evaluation

The PTV coverage, volumes of involved healthy tissue, conformity index (CI) [21] and conformation number (CN) [22], tumour control probabilities (TCP) and normal tissue complication probabilities (NTCP) have been calculated and compared between different radiotherapy approaches.

Prescription doses were established according to the BED calculated with an *α*/*β* value of 10 Gy.

We compared the boost treatments in terms of dosimetric and radiobiological features. We also evaluated the TCP and NTCP values as complete treatments (3D-CRT plus ICBRT boost versus IMRT pelvis plus boost) in order to test an ‘adding dose’ method.

### Dosimetric evaluations

We have converted both HDR and PDR ICBRT doses to their biological equivalents at 2 Gy as described in the following radiobiological parameters description. Moreover, the doses delivered by 3D-CRT and ICBRT were added together by using a modified ‘parameter adding’ method [23]. The dose distribution sum was compared to the one obtained with the IMRT technique.

To evaluate the PTV dose coverage, the percentage of volume receiving 95% and 85% of the prescription dose (V_95%_ and V_85%_) and the maximum and minimum doses were used. To compare the normal tissue doses, the percentage of rectum and urinary bladder volumes receiving 40 Gy (V_40Gy_) and 50 Gy (V_50Gy_), the V_40Gy_ for bowel and V_30Gy_ for spinal cord were used. Moreover, the value of D_2cm^3^_, defined as the minimum dose value in a volume of 2 cm^3^ receiving the highest dose [23] in the boost treatment, was calculated for urinary bladder and rectum. The doses to the normal tissues were compared in terms of equivalence to 2 Gy per fraction dose with an *α*/*β* value equal to 3 Gy.

To evaluate the quality of the different treatment approaches, the Radiation Therapy Oncology Group (RTOG) CI [24] and the CN [25] have been used.

The two parameters were defined as follows:

(1) $$Cl=\frac{TV_{Rl}}{TV}$$

(2) $$CN=\frac{\left(\frac{TV_{RI}}{TV}\right)}{\left(\frac{TV_{RI}}{V_{RI}}\right)}$$

where* TV_RI_* is the target volume encompassed by the prescription isodose, *TV* is the target volume and *V_RI_* is the volume encompassed by the prescription isodose; all volumes are expressed in cm^3^.

A CI value > 1 indicates that the irradiated volume is greater than the target volume and includes healthy tissues. On the other hand, a CI value < 1 indicates the target volume is only partially irradiated [21].

The value of CN is comprised between 0 and 1; a value of 1 represents a reference isodose covering exactly the target volume without irradiation of healthy tissue and indicates optimal conformation while a value of 0 means no conformation at all.

The CI indicator refers to the target itself while CN takes into account both target and healthy tissue irradiation [22].

In Table 3, the CI and CN values are reported.

### Radiobiological parameters: BED, TCP and NTCP

According to the literature, IMRT boost prescription doses were established calculating the BED of the ICBRT boost taking into account the dose rate [26–29] as follows:

(3) $$BED=D\left\{1+\left(\frac{2D}{\mu T\alpha /\beta }\right)*\left[1-\left(1/\mu_{T}\right)\left(1-e^{-\mu \tau }\right)\right]\right\}$$

and

(4) $$\mu =\frac{\ln2}{T_{1/2}}$$

where *D* is the total dose, *α* and *β* are the parameters of the linear-quadratic model and T_1/2_ is the half-time for damage repair, being repair rates (T_1/2_) and *α/β* ratios the main parameters which influence tissue responses when the dose rate is changed [29, 30]. The value of the parameters *α/β* was set equal to 10 Gy for tumour and 3 Gy for healthy tissue [31] and T_1/2_ was 1 hour for tumour and 3 hours for normal tissues [30, 32–36].

In Table 4, biological equivalent boost doses calculated according to eq. (3) for the tumour are reported.

From differential dose-volume histograms (DVHs), TCP and NTCP for rectum, urinary bladder wall and bowel were calculated, according to the Poisson [33] and Lymann–Burman’s [37, 38] models, respectively. The used equations were as follows:

(5) $$\mathrm{TCP}=e^{-N•e^{-(\alpha +\beta •\alpha )•D}}$$

where *α* and *β* are the radiobiological parameters, *D* is the total dose, d is the dose per fraction and *N* is the clonogenic cell number obtained setting the cell density equal to 10^5^ and 10^7^ cm^-3^ for patients that underwent postoperative and radically radiation treatment, respectively [39].

(6) $$\mathrm{NTCP}=\frac{1}{\sqrt{2\pi }}\int_{-\infty }^{x}e^{-\frac{x^{2}}{2}}dx$$

where $t=\frac{D-TD_{50}(v)}{m•TD_{50}}$ and $TD(v)=TD(1)•v^{-n}$

*v* is the fractional volume receiving a dose *D*, *TD* is the tolerance dose and *TD*_50_ is the tolerance dose corresponding to a 50% probability of complications to the healthy tissues, m and n are the parameters of the model. The values of the parameters *m*, *n* and *TD*_50_ were chosen equal to 0.14, 0.13 and 81 Gy, respectively, according to Peeters [40].

Table 5 reports the TCP and NTCP values for rectum, urinary bladder and bowel [41]. For EQD2 calculation, the American Brachytherapy Society worksheet [41] was used (available at https://www.americanbrachytherapy.org/resources/for-professionals/physics-corner/). Tables 6 and 7 summarise the EQD2 values.

## Results

### Boost by ICBRT versus IMRT

As expected, IMRT boost dose distribution was more homogeneous than ICBRT, in fact the comparison was statistically significant in terms of maximum and minimum dosees (*p* = 0.001 and *p* < 0.0001, respectively), V_85Gy_ and V_95Gy_ (*p* = 0.0002 and *p* = 0.0001, respectively), and CN and CI (*p* < 0.0001 and *p* = 0.0003, respectively). When comparing OARs doses, the value of D_2cm^3^_ was in favour of ICBRT. In fact, the D_2cm^3^_ values were lower for ICBRT than IMRT, being the mean value of the prescription dose for rectum, bowel and urinary bladder of 47.95%, 39.12% and 30.95% for ICBRT and 89.54%, 91.15% and 78.57% for IMRT, respectively.

Analysing TCP and NTCP values, the whole treatment IMRT was relatively superior. However, these results should be taken into account only for the IMRT part, due to the meaningless comparison between 3D-CRT and IMRT for whole pelvis treatment. In fact, the high TCP and low NTCP values of the IMRT analysis can confirm the feasibility of this approach when the ICBRT one is not available. The EQD2 results were similar, by virtue of our study design.

## Discussion

In this study, the radiobiological and dosimetric features of the boost treatment for gynaecological cancer have been analysed: the boost delivered by IMRT was compared to the boost delivered by ICBRT. Even though each technique presents the pros and cons, our results showed that both standard and IMRT approaches theoretically allow treating gynaecological cancer in a safe and effective way. Although it would be interesting to compare the results of postoperative and exclusive settings, the small number of studied patients does not allow a meaningful analysis. Moreover, this study aimed to evaluate only the feasibility of the radiation boost treatment for gynaecological tumours with IMRT in terms of radiobiology and dosimetry and to test a rigorous method for related evaluations. For those reasons, we have chosen a heterogeneous population: both cervical and endometrial patients, post-operative treatments and definitive treatments.

According to Gynaecological Groupe Européen de Curiethérapie-European Society for Therapeutic Radiology and Oncology [(GYN)GEC-ESTRO] working group (II) [42], cumulative DVHs are used for evaluation of the dose heterogeneity.

In gynaecological cancer, the treatment planning and the doses that can be prescribed are significantly influenced by the location of the OARs. Since OARs are close to the brachytherapy sources, only sigmoid bowel instead of the entire bowel is taken into account, besides rectum and urinary bladder. The GTV and CTV in brachytherapy are indeed close to the sources, usually within 15–40 mm, and are dependent on the position of the applicator, size and location of the tumour and cervix. Due to the steep fall-off of the dose close to the sources, there is a significant change in dose and dose-rate throughout the target volumes. The closer to the source, the more pronounced this effect: the dose along an axis perpendicular to the intrauterine source at the level of point A decreases from approximately 200% to 100% of the dose to point A when going from 10 to 20 mm from the source, whereas dose decreases from 100% to approximately 60% from 20 to 30 mm [42].

Notably, the gradient might be even steeper in terms of biologically equivalent dose, since not only dose but also dose rate follow this gradient effect. This dose inhomogeneity is certainly of major importance for the biological effect of brachytherapy. Therefore, even though IMRT provides a more homogenous dose distribution, this aspect could not be an advantage for tumour control, taking into account that a physical dose delivered by ICBRT could not yield the same biological effects. Moreover, when applying IMRT in place of ICBRT, the crucial key should be the prescription of the dose to the high-risk CTV (CTV-HR) [43, 44] as heterogeneous as possible, in order to copycat brachytherapy dosimetry. This could allow delivering more than 80–85Gy EQD2 to CTV-HR [43, 44].

Important to note, when comparing OARs doses, the value of D_2cm^3^_ was always lower for ICBRT respect to IMRT. Several investigators [45, 46] compared dosimetric parameters of the entire external OARs volumes with those of only the OARs wall volumes both for urinary bladder and rectum. When the volume of 2 cm^3^ is considered, the D_2cm^3^_ values computed for the external contours are almost identical to the D_2cm^3^_ values for the organ wall. In such cases, this implies that volumes receiving the highest doses are situated entirely within the organ wall. These data resulted in the designation of a new maximal small-volume dose by the gynaecological (GYN)GEC-ESTRO working group for image-guided brachytherapy, where the D_2cm^3^_ value was designated as the surrogate for a ‘hot spot’ for OARs [42, 47].

The NTCP calculated for the whole treatment by performing the DVH ‘parameter adding’ method resulted lower in the case of IMRT for all OARs, thus raising the question whether D_2cm^3^_ is a representative parameter as a predictor of toxicity [23]. In fact, IMRT will certainly give a very high dose to a small volume of the organ but significant correlation with radiation-induced toxicity have been found mostly with the intermediate-high dose part of the DVH [48].

Interestingly, our predicted results are in agreement with the clinical findings reported by other authors [16] taking into account that the crude rate of incidence of toxicity involves also the effect of chemotherapy; nevertheless, they found that IMRT was associated with low rates of acute and late high-grade toxicity and that outcomes were comparable to those expected with conventional techniques.

Several retrospective cohort studies [4] have provided support for the use of IMRT over conventional techniques but some concern should be considered. First, regarding the potential increasing risk of second malignancies due to the low-dose irradiation of normal tissues [49]. Second, IMRT planning needs to take into consideration both organ motion and tumour regression, as several studies have reported on the inter-fraction and intra-fraction motions of the cervix [50–55]. In fact, Haripotepornkul *et al* [56] in their study concluded that daily image guidance and possibly re-planning of the treatment volumes are necessary to improve the accuracy of IMRT by accounting for the unpredictable changes in cervical position. Finally, quite large margins that are necessary for IMRT planning might lead to unnecessary overdosing of healthy tissues while the tumour shrinks as a consequence of the therapy. Moreover, uncertainties due to intrafraction variations can contribute to the treatment-related late side [57]. It should be taken into account that as ICBRT necessitates a dedicated brachytherapy unit and costly periodic source changes, IMRT should be applied with a brachytherapy-like immobilisation system and irradiation should be always image-guided delivered. Even though the theory comparing IMRT to ICBRT can demonstrate similar target radiation coverage, the results of clinical studies are certainly more significant. Recently, Holschneider *et al* [58] published a literature review about brachytherapy for cervical cancer. The authors conclude that the Society of Gynecologic Oncology and the American Brachytherapy Society concur with National Comprehensive Cancer Network (NCCN) guidelines that conformal external beam therapies such as IMRT or stereotactic radiotherapy should not be used as alternatives to brachytherapy in patients undergoing primary curative-intent radiation therapy for cervical cancer [58]. As a matter of fact, the present study has been designed precisely for those patients who cannot be treated with brachytherapy for clinical or personal reasons. Sure enough, IMRT can be exploited to deliver simultaneous integrated boost, thereby reducing the overall treatment time. This overall treatment time reduction might be an advantage not only for the quality of life of the patients but also for increasing tumour control, thus becoming more similar to the low conformed ICBRT boost dose. Moreover, this aspect could increase the treatment tolerance for elderly patients and could reduce the number of older undertreated patients [59].

This study presents some limitations, the most important being heterogeneity and small number of the analysed patients and lack of clinical follow-up data. Furthermore, because we analysed a population of patients treated before 2006, we cannot carry out any comparison to the recent GEC-ESTRO GYN working group paper [60] that defines the current approach to brachytherapy for gynaecological cancers. In fact, advanced EBRT like IMRT has already considered the techniques of choice for these treatments [60].

However, this study defines the pros and cons of advanced EBRT also in place of ICBRT when brachytherapy is unattainable. Both elderly patients and patients with anatomical conformation unsuitable for ICBRT should receive a whole treatment schedule without dose reduction or target missing.

## Conclusions

More addressed and prospective studies are needed to draw any clear conclusion, though this study confirms that IMRT can provide a valid approach for cervical and endometrial cancer treatment as an alternative to standard radiation therapies when brachytherapy is not feasible.

## Conflict of interest

Nothing to declare.

## Funding statement

This research did not receive any specific grant from funding agencies in the public, commercial, or not-for-profit sectors.

## Figures and Tables

**Table 1.** Patients and baseline disease characteristics.

Evaluable patients, *n* = 15	
**Mean age at diagnosis (range), years**	**48.4 (30–76)**
**Tumour Histology** – SCC – Adenocarcinoma – Endometrioid – Mucinous – Adenosquamous	**Number (%)** 9 (60) 2 (13) 2 (13) 1 (7) 1 (7)
**Grading** – G1 – G2 – G3 – NA	0 (0) 3 (20) 6 (40) 6 (40)
**Staging (FIGO)** – IA – IB1 – IB2 – IIA – IIB – IIIA – IIIB	1 (7) 4 (27) 5 (33) 1 (7) 3 (19) 0 (0) 1 (7)
**Evaluable patients, *n* = 10**	
**Pathological T staging** – pT1a – pT1b – pT2a – pT2b – Recurrence	**Number (%)** 0 (0) 4 (40) 0 (0) 5 (50) 1 (10)
**Pathological N staging** – pNx – pN0 – pN1	1 (10) 3 (30) 6 (60)

N, nodal; NA, not available; SCC, squamous cell carcinoma; T, tumour

**Table 2.** OARs and target volumes.

	Volume ± σ (cm^3^)	Range (cm^3^)
**Target**		
PTV pelvis	1291.29 ± 184.26	1051.40–1765.96
ICBRT boost	37.04 ± 20.25	11.99–87.60
IMRT boost	94.51 ± 60.41	36.10–244.70
**Rectum wall**		
ICBRT boost	21.45 ± 9.11	9.06–48.47
IMRT boost	23.42 ± 10.37	11.80–50.60
**Bladder wall**		
ICBRT boost	29.86 ± 8.15	15.30–45.84
IMRT boost	31.54 ± 20.30	9.10–67.40

OARs, organs at risk; PTV, planning target volume; ICBRT, intra-cavitary brachytherapy; IMRT, intensity-modulated radiation therapy

**Table 3.** CI and CN for the different treatments.

	Mean Value ± σ (%)	Range (%)
CI		
3D-CRT-pelvis	0.99 ± 0.02	0.91–1.00
IMRT- pelvis	0.96 ± 0.02	0.93–0.99
ICBRT- boost	0.73 ± 0.17	0.35–0.91
IMRT- boost	0.97 ± 0.01	0.95–0.99
CN		
3DCRT- pelvis	0.47 ± 0.05	0.39–0.55
IMRT- pelvis	0.85 ± 0.03	0.80–0.88
ICBRT-boost	0.24 ± 0.15	0.03–0.55
IMRT- boost	0.78 ± 0.10	0.62–0.90

3D-CRT, 3-dimensional conformal radiotherapy; IMRT, intensity-modulated radiation therapy; ICBRT, intra-cavitary brachytherapy

**Table 4.** Treatment boost: BED for tumour (α/β = 10 Gy).

ICBRT treatment		IMRT treatment	
HDR 5 Gy × 3 = 15 Gy	BED = 22.5 Gy	1.8 Gy × 11 fr = 19.8 Gy	BED = 23.4 Gy
PDR 15 Gy (eq to HDR 4 Gy × 3 = 12 Gy)	BED = 16.8 Gy	1.8 Gy × 8 fr = 14.4 Gy	BED = 17.0 Gy
PDR 30 Gy (eq to HDR 4 Gy × 6 = 24 Gy)	BED = 33.6 Gy	1.8 Gy × 16 fr = 28.8 Gy	BED = 34.0 Gy

ICBRT, intra-cavitary brachytherapy; IMRT, intensity-modulated radiation therapy; HDR, high dose rate; PDR, pulsed dose rate

**Table 5.** TCP and NTCP for OARs and target.

	Mean ± σ (%) 3D-CRT plus ICBRT	Mean ± σ (%) IMRT
TCP boost	80.13 ± 25.10	99.51 ± 0.88
NTCP rectum wall	27.98 ± 44.38	4.62 ± 5.64
NTCP bladder wall	1.46 ± 3.54	0.09 ± 0.13
NTCP bowel	8.95 ± 7.95	6.09 ± 3.84

TCP, tumour control probability; NTCP, normal tissue control probability; 3D-CRT, 3-dimensional conformal radiotherapy; IMRT, intensity-modulated radiation therapy; ICBRT, intra-cavitary brachytherapy

**Table 6.** Treatment boost: equivalent biological dose delivered in 2 Gy fractions (EQD2) for tumour (*α*/*β* = 10 Gy).

ICBRT treatment		IMRT treatment	
HDR 5 Gy × 3 = 15 Gy	EQD2 = 18.8 Gy	1.8 Gy × 11 fr = 19.8 Gy	EQD2 = 19.5 Gy
PDR 15 Gy (eq to HDR 4 Gy × 3 = 12 Gy)	EQD2 = 14.2 Gy	1.8 Gy × 8 fr = 14.4 Gy	EQD2 = 14.2 Gy
PDR 30 Gy (eq to HDR 4 Gy × 6 = 24 Gy)	EQD2 = 28 Gy	1.8 Gy × 16 fr = 28.8 Gy	EQD2 = 28.3 Gy

ICBRT, intra-cavitary brachytherapy; IMRT, intensity-modulated radiation therapy; HDR, high dose rate; PDR, pulsed dose rate

**Table 7.** EQD2 for tumour (*α*/*β* = 10 Gy).

	Mean ± σ (%) 3D-CRT plus ICBRT	Mean ± σ (%) IMRT
EQD2	70 ± 2.5	69.9 ± 4

EQD2 equivalent biological dose delivered in 2 Gy fractions; 3D-CRT, 3-dimensional conformal radiotherapy; IMRT, intensity-modulated radiation therapy; ICBRT, Intra-cavitary brachytherapy
